# Supplementary material for: Prognosis of Atrial Fibrillation with or without Comorbidities: Analysis of Younger Adults from a Nationwide Database
Source: J Clin Med. 2022 Apr 1;11(7):1981. doi: 10.3390/jcm11071981 (PMC8999868; doi:10.3390/jcm11071981)
Supplement: Supplementary file 1 [file jcm-11-01981-s001.zip › jcm-1652434-supplementary.pdf]

Supplementary Figure S1. Atrial fibrillation patients with age < or >60 with "Lone" AF, AF with known cardiac disease and AF with extra-cardiac disease (AF: Atrial fibrillation, KCD : known cardiac disease, ECC: extra-cardiac comorbidities).

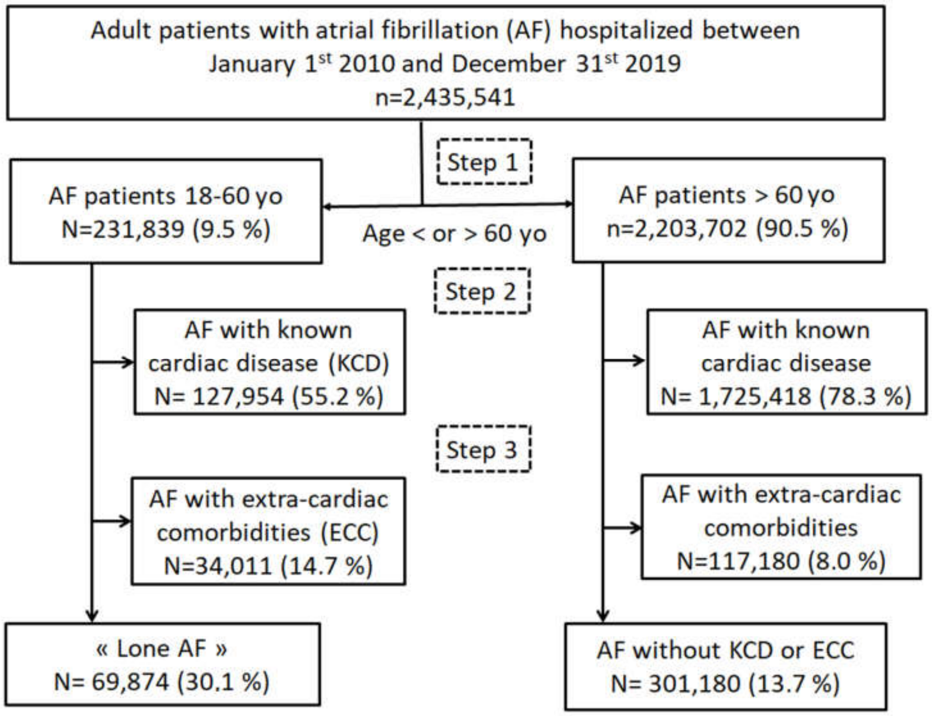

Supplementary Figure S2. Cumulative incidences for all-cause death (top panel) or cardiovascular death (middle panel) and non-cardiovascular death (lower panel) during follow-up in patients with atrial fibrillation (KCD : known cardiac disease, ECC : extra-cardiac comorbidities).

All-cause death

≤ 60 years old **> 60 years old**

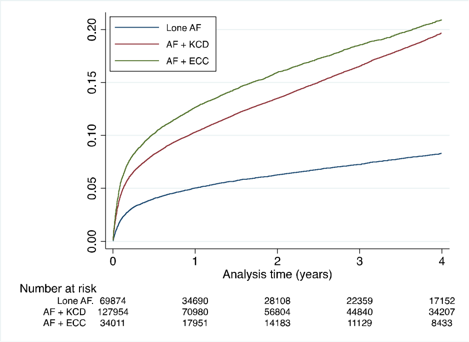

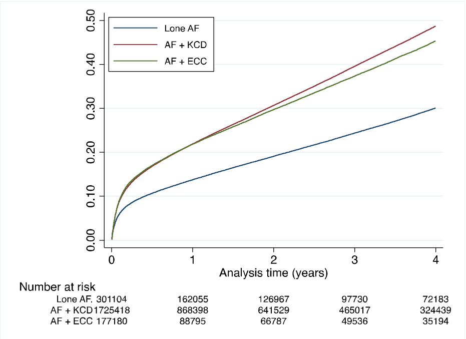

Cardiovascular death

≤ 60 years old **> 60 years old**

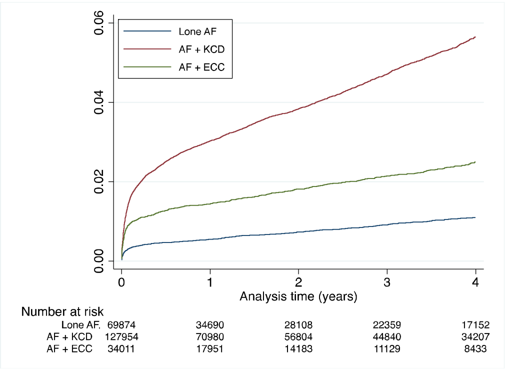

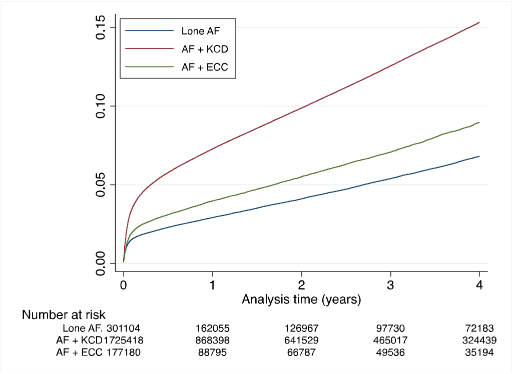

Non-cardiovascular death

≤ 60 years old **> 60 years old**

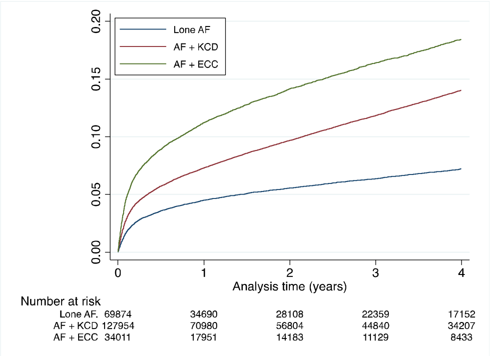

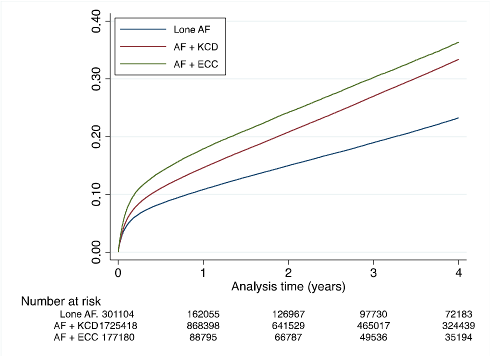

Supplementary Figure S3. Cumulative incidences for ischemic stroke (top panel) or rehospitalization for heart failure (HF) (lower panel) during follow-up in patients with atrial fibrillation (KCD: known cardiac disease : ECC: extra-cardiac comorbidities).

Ischemic stroke

≤ 60 years old

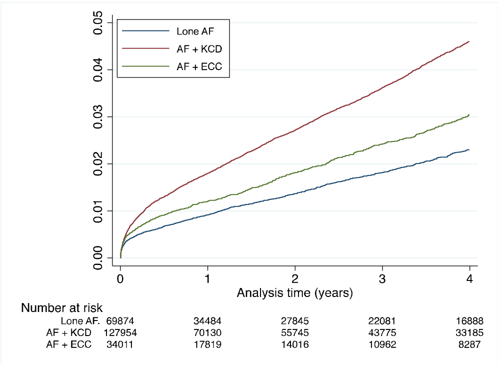

> 60 years old

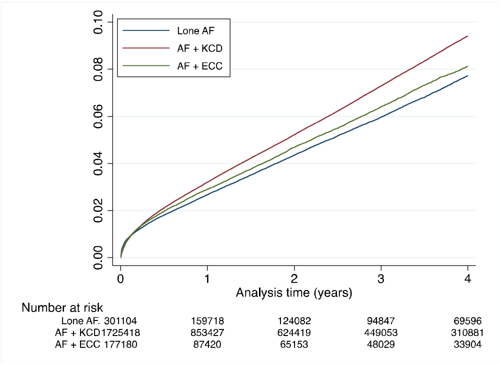

Hospitalization for heart failure

≤ 60 years old

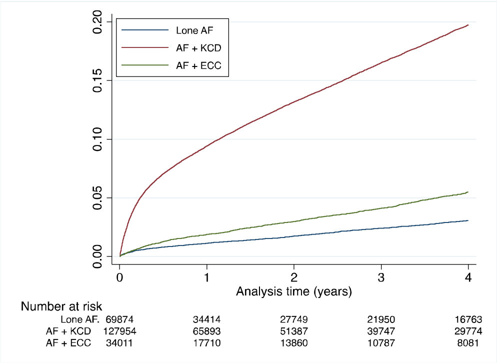

> 60 years old

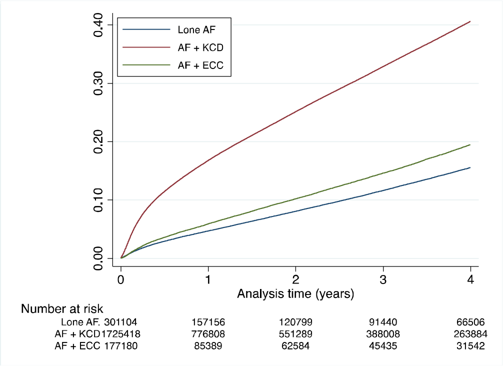

Supplementary Table S1. Baseline characteristics of adult patients hospitalized in France (2011-2020) with history of AF.

	**Age ≤ 60 (*n*=231,839)**	**Age > 60 (*n*=2,203,702)**	**p**	**Total (*n*=2,435,541)**
Age (years), mean±SD	51.8±8.4	79.9±8.9	<0.0001	77.2±12.1
Sex (male), *n (%)*	167,893 (72.4)	1,118,420 (50.8)	<0.0001	1,286,313 (52.8)
Hypertension, *n (%)*	79,398 (34.2)	1,379,667 (62.6)	<0.0001	1,459,065 (59.9)
Diabetes mellitus, *n (%)*	33,565 (14.5)	476,052 (21.6)	<0.0001	509,617 (20.9)
Smoker, *n (%)*	42,942 (18.5)	119,773 (5.4)	<0.0001	162,715 (6.7)
Dyslipidemia, *n (%)*	38,179 (16.5)	486,310 (22.1)	<0.0001	524,489 (21.5)
Obesity, *n (%)*	44,952 (19.4)	302,044 (13.7)	<0.0001	346,996 (14.2)
Heart *failure* with congestion, *n (%)*	59,249 (25.6)	823,181 (37.4)	<0.0001	882,430 (36.2)
History of pulmonary edema, *n (%)*	8188 (3.5)	52,236 (2.4)	<0.0001	60,424 (2.5)
Mitral regurgitation, *n (%)*	13,036 (5.6)	129,962 (5.9)	<0.0001	142,998 (5.9)
Mitral stenosis, *n (%)*	1463 (0.6)	4569 (0.2)	<0.0001	6032 (0.2)
Aortic regurgitation, *n (%)*	5399 (2.3)	54,315 (2.5)	0.0001	59,714 (2.5)
Aortic stenosis, *n (%)*	6352 (2.7)	143,440 (6.5)	<0.0001	149,792 (6.2)
Previous endocarditis, *n (%)*	2067 (0.9)	8670 (0.4)	<0.0001	10,737 (0.4)
Dilated cardiomyopathy, *n (%)*	23,809 (10.3)	145,299 (6.6)	<0.0001	169,108 (6.9)
Coronary artery disease, *n (%)*	42,025 (18.1)	554,684 (25.2)	<0.0001	596,709 (24.5)
Previous MI, *n (%)*	12,445 (5.4)	106,785 (4.8)	<0.0001	119,230 (4.9)
Previous PCI, *n (%)*	10,238 (4.4)	87,003 (3.9)	<0.0001	97,241 (4.0)
Previous CABG, *n (%)*	4853 (2.1)	74,969 (3.4)	<0.0001	79,822 (3.3)
Vascular disease, *n (%)*	30,992 (13.4)	397,534 (18.0)	<0.0001	428,526 (17.6)
Left BBB, *n (%)*	2820 (1.2)	47,001 (2.1)	<0.0001	49,821 (2.0)
Right BBB, *n (%)*	3241 (1.4)	51,174 (2.3)	<0.0001	54,415 (2.2)
Previous pacemaker or ICD, *n (%)*	5009 (2.2)	89,682 (4.1)	<0.0001	94,691 (3.9)
Ischemic stroke, *n (%)*	9186 (4.0)	164,377 (7.5)	<0.0001	173,563 (7.1)
Intracranial bleeding, *n (%)*	3178 (1.4)	49,321 (2.2)	<0.0001	52,499 (2.2)
Alcohol-related diagnoses, *n (%)*	28,451 (12.3)	93,767 (4.3)	<0.0001	122,218 (5.0)
Abnormal renal function, *n (%)*	7151 (3.1)	159,779 (7.3)	<0.0001	166,930 (6.9)
Lung disease, *n (%)*	28,981 (12.5)	374,278 (17.0)	<0.0001	403,259 (16.6)
Sleep apnea syndrome, *n (%)*	16,188 (7.0)	110,912 (5.0)	<0.0001	127,100 (5.2)
COPD, *n (%)*	15,804 (6.8)	213,082 (9.7)	<0.0001	228,886 (9.4)
Liver disease, *n (%)*	14,342 (6.2)	74,400 (3.4)	<0.0001	88,742 (3.6)
Thyroid diseases, *n (%)*	14,411 (6.2)	218,819 (9.9)	<0.0001	233,230 (9.6)
Inflammatory disease, *n (%)*	9179 (4.0)	136,352 (6.2)	<0.0001	145,531 (6.0)
Anemia, *n (%)*	24,523 (10.6)	389,687 (17.7)	<0.0001	414,210 (17.0)
Previous cancer, *n (%)*	24,336 (10.5)	403,937 (18.3)	<0.0001	428,273 (17.6)
CHA2DS2VASc score, mean±SD	1.2±1.2	3.7±1.5	<0.0001	3.5±1.6
HASBLED score, mean±SD	1.1±1.2	2.5±1.1	<0.0001	2.4±1.2
Charlson comorbidity index, mean±SD	2.3±2.8	3.6±2.8	<0.0001	3.5±2.8
Frailty index, mean±SD	4.1±6.0	9.8±9.3	<0.0001	9.3±9.2

BBB = bundle branch block; CABG = coronary artery bypass graft; COPD = chronic obstructive pulmonary disease; ICD = implantable cardioverter defibrillator; MI = myocardial infarction; PCI=percutaneous coronary intervention.
